# Exploring experiences of ageing in older adults living with HIV in Sweden: a qualitative study

**DOI:** 10.1080/17482631.2024.2393752

**Published:** 2024-08-27

**Authors:** Annelie J. Sundler, Marie Lund, Sandra van Dulmen, Ewa Carlsson Lalloo

**Affiliations:** a Faculty of Caring Science, Work Life and Social Welfare, University of Borås, Borås, Sweden; b Department of Communication in Healthcare, Nivel, Netherlands Institute for Health Services Research, Utrecht, The Netherlands; c Radboud Institute for Health Sciences, Department of Primary and Community Care, Radboud University Medical Center, Nijmegen, The Netherlands

**Keywords:** Human immunodeficiency virus, older adults, qualitative, health experiences, ageing, care

## Abstract

**Purpose:**

The number of people living and ageing with HIV is increasing. Insight into their well-being is lacking. The present study was conducted to explore needs, psychosocial issues and experiences of ageing in older adults living with human immunodeficiency virus (HIV) in Sweden.

**Methods:**

A qualitative approach was employed. Semi-structured interviews were conducted with a convenience sample of 22 participants aged 65 years or older living with HIV in Sweden. Thematic analysis based on descriptive phenomenology was used.

**Results:**

Being an older adult living with HIV emerged as a multifaceted experience. The meaning and impact of HIV varied from minimal to substantial, and ageing could overshadow the significance of HIV in everyday life. Three themes emerged: 1) increasing age in the foreground, 2) internalizing HIV in everyday life, and 3) socioemotional impact on everyday life.

**Conclusions:**

The findings suggest a need for a more holistic approach to care of older adults living with HIV, recognizing the broader context of healthy ageing. While participants experienced good health, challenges related to ageing persist, and experiencing HIV-related stigma. The findings highlight the importance of empowering older adults living with HIV.

## Introduction

The effectiveness and accessibility of antiretroviral treatment (ART) have prolonged the life expectancy of people living with human immunodeficiency virus (HIV), which can now be considered a chronic infection (Lohse et al., 2011). The global proportion of people aged 50 and older living with HIV increased from 8% in year 2000 to 16% in 2016 and was expected to reach 21% by year 2020 (Autenrieth et al., 2018). This has led to an increase in the number of older adults, aged 65 and above, living and ageing with HIV. However, living with HIV as an older adult can be challenging because of the higher incidence of comorbidities compared to the rest of the population in the same age group (Kong et al., 2019; Pourcher et al., 2020). Additionally, older adults living with HIV are at risk of being exposed to multiple stigmatizations (Emlet, 2006), which entails encountering negative attitudes or moral judgements from others when a person breaks perceived socially accepted norms (Goffman, 1990), such as the expectation that older adults should not be sexually active. HIV-related stigma can negatively affect quality of life and well-being (The Public Health Agency of Sweden, 2016), and older adults can encounter both HIV-related stigma and ageism, which can lead to discrimination. Still, research on subjective experiences of older adults living with HIV is scarce.

In Sweden, approximately 8,300 individuals lived with HIV in 2022, of which 22% are aged 60 years or older and 7% are aged 70 years or older (Carlander & Mattsson, 2023). Most individuals living with HIV (98%) in Sweden are on ART, of whom 95% have well-adjusted HIV treatment with undetectable viral loads (Carlander & Mattsson, 2023). Sweden was the first country in the world to meet the Joint United Nations Programme on HIV/AIDS (UNAIDS) 90-90-90 targets (Gisslén et al., 2017), meaning that 90% of all people living with HIV must be diagnosed, 90% of these must be treated with ART, and 90% of these must have undetectable virus level (UNAIDS, 2020). Without detectable virus levels, there is no risk of transmitting HIV sexually, described as HIV being “undetectable” = “untransmittable” (U=U) (Cambiano et al., 2019). As a result of increased survival and better global access to ART, the targets have been raised to 95%, including a fourth target proposing that 95% of those living with HIV should have a good health-related quality of life and zero discrimination (UNAIDS, 2024). Thus, there has been a shift to focus on how HIV impacts individuals’ quality of life (Lazarus et al., 2016), including balancing the psychosocial aspects of living with HIV with the demands on medical issues connected to treatment and symptoms.

Ageing affects health; healthy ageing is described as the ability to continue developing, being part of a social context, having abilities, and experiencing meaningfulness and autonomy (World Health Organization, 2021). Health and well-being are crucial for several reasons and can contribute to an individual’s overall quality of life. Health does not necessarily mean the absence of a disease, as a person can experience well-being and good health despite illness (World Health Organization, 1948). Understanding the complexities of health calls for an existential view in which health can be related to meaningfulness and capacity in life (Dahlberg et al., 2009). The individual constitutes health, which can change over time and depend on context (Arman et al., 2015). Health is a resource in everyday life and includes a sense of vitality to take advantage of life’s opportunities and the time we live until death (Gadamer, 1996). Therefore, it is vital to have a holistic view of health and not only focus on the medical aspects of individuals living with HIV.

Due to the development of ART and prolonged life expectancy there is a growing population of older adults living and ageing with HIV. Hitherto, a cut-off age of 50 years is used to define older age in adults living with HIV. This is based on evidence suggesting poorer immunological recovery, increased risk of comorbidities, and an impact on survival among older individuals living with HIV (Sánchez-Conde et al., 2019). However, there is a notable gap in research focusing on experiences of older adults aged 65 years and above living with HIV and insight into their experiences, specific needs and psychosocial aspects of importance when ageing. Therefore, this study aimed to explore lived experiences with ageing in adults aged 65 years or older living with HIV in Sweden.

## Materials and methods

### Design

A qualitative study with semi-structured interviews was conducted. Data were analysed with thematic analysis as described by Sundler et al. (2019), based on descriptive phenomenology. The Consolidated Criteria for Reporting Qualitative Research (COREQ) was used to report the quality of the research (Tong et al., 2007).

### Phenomenology—ontological and epistemological approach

This study was grounded in descriptive phenomenology originating from Husserl and further developed by Merleau-Ponty (Dahlberg et al., 2008) based on the notion that attention needs to be paid to the subjective and lived body in health and care. Phenomenology is the study of phenomena that appear as humans experience them. The lifeworld perspective becomes central and can be understood as a world with meanings in which individuals experience and share their experiences to understand lived experiences (Dahlberg et al., 2008). The lifeworld is an intersubjective world accessed through the lived body, and there is no thinking that is separate from the body. The body, subject, and the world are understood as intertwined (Dahlberg et al., 2008; Merleau-Ponty, 1945/1962). This understanding of the lifeworld and human intersubjectivity has implications for the research process, that is, data collection and analysis.

The methodological principles of thematic analysis include an approach in which researchers strive for openness, questioning pre-understandings, and a reflective attitude. Openness is reflecting on preconceptions and judgements of the world and our experiences. Examining one’s pre-understanding and critical reflection help the researcher become aware of their natural attitude and process of understanding the data. These principles guide the researcher during the research process to allow sensitivity and openness to the complexity and uniqueness of an individual’s experiences (Sundler et al., 2019).

### Sampling and participants

A convenience sample of older adults living with HIV was recruited for this study. Participants eligible for inclusion were people living with HIV, aged 65 years and older, speaking English or Swedish. Exclusion criteria were people diagnosed with HIV less than six months ago or individuals with a diagnosis of dementia. To capture the diversity of participants’ experiences with the explored phenomenon, they were also selected based on factors such as country of origin, gender and residence.

Recruitment for the study was carried out from September 2022 to December 2022. Information regarding the study was provided through digital forums and at all Swedish HIV clinics for adults. An informative post about the study was placed on the official website of the University of Borås. Information was also distributed by civil society organizations supporting people living with HIV. At HIV clinics, nurses, counsellors, and physicians identified eligible participants and provided them with information about the study and invitations to participate. Participants could either contact the researchers themselves, or the clinicians handed over their contact information to the researcher. Three participants contacted the researchers themselves. When talking with possible participants, the researchers again gave them information about the study and a chance to ask questions. Among potential participants 29 initially were interested in the study, three of them didn’t respond when the researchers tried to contact them and four declined further participation after receiving more information about the study. In total, 22 participated in the interviews. The number of individuals asked for interest in participating in the study was not recorded.

### Data collection

Individual semi-structured interviews were conducted to capture their experiences of the phenomenon. A semi-structured interview guide with open-ended questions was developed and supplemented with follow-up questions. The interview guide was discussed and created with reference persons from civil society and HIV organizations. The interview questions were evaluated by the research team after the first two interviews and found to be effective, thus no changes were made. In exploring their experiences of living with HIV and ageing, the participants were asked to describe their experiences. The interviewer listened carefully and followed up on topics that appeared with questions such as “Can you give an example?” or “Can you elaborate on this?”. Field notes were not taken. Demographic information was voluntary to answer and was not recorded but documented by the researcher.

The participants chose to be interviewed onsite at an agreed-upon location or via telephone. Most of the interviews (*n* = 19) were conducted via telephone by ML and ECL. Reasons for choosing to be interviewed via telephone were not asked for, but several of the participants expressed that it was more convenient to be interviewed via telephone The interviews lasted from 25 min to 1 h and 27 min, with an average duration of 49 min. The interviews were conducted by the researchers in Swedish, audio recorded, and transcribed verbatim.

### Data analysis

The analysis followed the thematic analysis steps (Sundler et al., 2019). Thematic analysis was used to understand and describe patterns in the meanings of the lived experiences of the studied phenomenon (Sundler et al., 2019). The analysis began with careful and repeated readings to obtain an overall sense of the data. The reading then shifts to focus on the meaning of the data. The identified meanings were compared for differences and similarities. From the patterns derived from the data, the text develops initial themes. The meanings of these themes were reviewed and described. The researchers reviewed, discussed, and refined emerging themes and results during analysis. The analysis was inductive, meaning the themes were data-driven (Sundler et al., 2019).

The researchers began by reading the transcribed interviews, in Swedish, with an open-minded awareness of possible pre-understanding. To become familiar and get an overall sense of experiences expressed by the participants, the interviews were read in their entirety. Next step was to search for meaning related to the aim of the study. The meanings were marked in the transcribed interviews and summarized in short notes. These notes were later used, along with quotes, to develop a descriptive text for the meanings found. The final step was to compare the similarities and differences between the described meanings and organize them into patterns. Based on these patterns, themes were developed to clarify the meanings of the lived experiences in the actual context. The researchers continuously reviewed and refined the themes developed during this process. The descriptive text and themes derived were written in English. Quotes from participants were used to illustrate the themes and meanings. A professional English-speaking native language editor reviewed the translated quotes. The quotes used were anonymized to avoid exposing the participants’ identities. The analysis was primarily performed by ECL, a female researcher with a background in HIV care and qualitative research experience, and ML, a female researcher. The analysis was further discussed and reviewed by AS and SvD, female researchers with extensive qualitative and thematic research experience.

### Ethical considerations

Ethical approval was obtained from the Swedish Ethical Review Authority (no. 2022-02,203-01). The research was conducted in accordance with the Declaration of Helsinki (World Medical Association WMA, 2018).

Participants received oral and written information about the study, including its voluntary nature and the option to withdraw without reason, ensuring their care would not be impacted. All participants gave oral or written consent, which was documented by researchers. No incentives were offered to the healthcare providers and HIV-organizations for providing information about the study and participants were not reimbursed for their participation.

## Results

### Characteristics of the study participants

In total, 22 individuals aged 65 years or older, with an average age of 75 years, living with HIV participated. Not all participants answered every question about demographics. We included a mixture of people, and overall, there was a geographic spread across Sweden, from the north to the south, both from urban and rural areas as well as with diverse sexual orientations and educational backgrounds. Of the participants 14 identified themselves as men and 8 as women. They had varying experiences in healthcare and life situations. The number of years lived with HIV varied from two years to over 30 years. All participants were on HIV treatment and represented more than ten different HIV-clinics. Of the 22 participants, six were born in another country than Sweden. None of the participants were coupled together. It was common to live alone for multiple reasons, such as being divorced or widowed. Not all participants wanted to disclose how they contracted HIV or their sexual orientation. In summary, there were variations among participants linked to age, gender identity, sexual identity, family situation, route of transmission, years lived with HIV, place of residence, migration experience, and socioeconomic status. When asked about the needs for elderly- or home-care services, no participants expressed this need.

### Themes describing the experiences of being an older adult living with HIV

Overall, the results revealed that being an older adult living with HIV was experienced as multifaceted. The intersection of age and HIV brought numerous layers to life, revealing multiple dimensions of experiences. HIV occupied different spaces in individuals’ lives and affected everyday life to varying degrees, ranging from minimal to substantial. For older adults living with HIV, the prominence of ageing and being older commonly overshadowed the significance of HIV in their everyday lives. Living with HIV required navigation in daily life, such as medication management. There were specific moments when HIV served as a reminder, and it had both emotional and social impacts on them and their relationships with others.

The results are further described in three themes: increasing age in the foreground, internalizing HIV in everyday life, and the socioemotional impact on daily life.

#### Increasing age in the foreground

This theme describes the overarching influence of ageing and being old, where the prominence of ageing and being old was related to emotions and concerns that overshadowed the significance of HIV itself, placing HIV in the background of everyday life. The process of growing old was experienced as the primary lens through which individuals perceived and navigated their ordinary lives, diminishing the relative importance of an HIV diagnosis. For example, one participant described being old and living with HIV as an iceberg. In particular, where HIV represented the visible tip, and beneath the surface lay the entirety of a person’s life, full of experiences and aspects beyond HIV, such as other health issues. Thus, HIV was described as something that was not the focus of ageing.

I guess I will die from something completely different than HIV. (19)

Ageing was experienced as something one could not escape and was seen as a natural part of life, not causing too much concern. Finding meaning in life was crucial to persons’ well-being. This was articulated as the ability to continue life projects, maintain and have a sense of control, and make decisions about one’s own life. This brought joy and optimism for the future, creating feelings of freedom and lust for living. Having experiences with former challenges in life could increase gratitude for ageing.

It is the most nice thing I have experienced… //Being human … Being old and having life skills … You have to relax and choose. I can do everything. I cannot become a brain surgeon, but I have no plans to do so. Otherwise, I can do whatever I want. The freedoms and privileges I have now. I have never had that before. (13)

Experiences of feeling needed or wanted by someone were significant in fulfilling life with meaning. This contributed to a higher quality of life and reduced feelings of loneliness. A sense of coherence and belonging were fostered through social relationships, whether with a pet, partner, family member, various organizations, relatives, or friends. Having few or strained relationships with family or friends could increase feelings of loneliness. For example, when migrating from another country and leaving loved ones like family and friends behind. One participant emphasized the importance of having close contact with children and grandchildren.

What is important to me, you know, are my children. (12)

During ageing, striving for good health, encompassing physical and mental well-being, was essential. Efforts to maintain body shape and manage different kinds of activities, such as physical exercise, being out in nature, or travelling, were examples of activities enhancing overall health and quality of life:

That I should still be able to move freely and engage in the little exercise routine that I still maintain, that is important. (2)

Socioeconomic stability was one expressed enabling factor for engaging in activities and maintaining independence. Additionally, continuing to work in various forms could foster a sense of being needed and provide positive mental and physical challenges. Autonomy and the ability to choose when to work could strengthen feelings of well-being. Being active and nurturing interests were also expressed as necessary.

When you retire, it is important to have interests. Interests and to read and acquire interest. Otherwise, the time may feel very slow. (1)

Ageing experiences included physical ailments that caused limitations in daily life. Signs of ageing were gradually emerging in various ways, affecting both body and mind. Imagining life without HIV was for some difficult, especially for individuals living with HIV for many years. It was known that HIV could cause specific health issues or amplify some physical symptoms of ageing. However, there was still uncertainty regarding how HIV and ageing were connected. Bodily changes in ageing could evoke feelings of melancholy and sorrow, even leading to depression and struggles in finding meaning in life.

Sometimes you feel that getting old is not fun. //It feels like it is a journey toward death. (14)

For some, ageing was intertwined with feelings of loneliness because of friends or family members who have passed away or having separated from their partners.

#### Internalizing HIV in everyday life

Internalizing HIV in everyday life entailed integrating living with it into various aspects of daily life. The experiences revealed a temporality in which past life experiences and life expectancies shaped the present moment. When HIV became internalized, there were moments when participants were reminded of its presence in their lives.

HIV was perceived as something that did not occupy a prominent space in daily life. Instead of focusing on the obstacles and challenges that HIV could present, it was considered essential to maintain a forward-looking and positive attitude about life and the future. Some experienced themselves as healthier and more energetic than those of the same age without HIV. For some participants, living with HIV had even brought meaning into their lives, and HIV-related challenges had made them grow as persons. There was a sense of “being able to prioritize what is important in life” (22). Intimate relationships and sexual life had improved for some.

Yes, because after the diagnosis, we had limitations, and then when the viral load or values had dropped, those limitations ceased. During this period, new solutions were obtained. Therefore, I can almost feel that we have at least a good sex life now, and almost better than when we were younger. (6)

One crucial aspect that enabled the participants to internalize HIV was an undetectable viral load, which assured them that HIV was no longer transmittable. With an undetectable viral load, HIV was experienced as less prominent in life, and a low viral load guaranteed a healthy life without any risk of viral transmission.

It is truly unbelievable that you can achieve this despite having such a serious condition. Its incredible that you can walk around here like anyone else, without any sign of it. (21)

However, past experiences from the period of receiving an HIV diagnosis, a tangible life-changing period in life, still affected some of the participants. Various experiences were described, and different thoughts and emotions were associated with HIV acquisition. For some, receiving a diagnosis was a shock or a feeling like receiving a death sentence. Some had received the diagnosis at a late stage, despite having sought medical help multiple times triggered by HIV-related symptoms such as fever and weight loss, indicating that the immune system was affected. Others found it difficult to believe that they had acquired HIV and thought that there had been a mistake.

After receiving an HIV diagnosis, older adults experienced a process that was described as going from thinking of HIV all the time to something one gets used to and internalizes.

It was challenging in the beginning, of course. //After the first year, I did not think that way. (6)

As a part of this process, receiving information about the effectiveness of HIV medication and acquiring knowledge about HIV, especially about the non-risk of HIV transmission, were described as critical. This was described as helping to cope with HIV and creating feelings of reassurance, bringing hope for future life. This information was mainly retrieved from HIV clinics but also from the Internet or others living with HIV.

Those receiving the diagnosis in the 80s/90s described experiences of ”surviving a death sentence” that for some made them feel empowered and contributed to new perspectives on life.

It seemed apparent that my days were being counted. However, there was a turnaround in medication by the end of the 90s. (22)

But for some it also raised a feeling of sadness for being alone as a survivor, experiences related to having seen people around them die of HIV.

Yes, well, now that I am very old, so many have died, almost everyone is gone. I am the only one left. And especially AIDS, everyone died from AIDS. I think about it; I live alone. I feel very lonely’. Nonetheless, I cannot break it, you know (19).

Even though HIV was internalized, the participants were reminded of its presence in daily life on specific occasions, such as when visiting an HIV clinic or taking HIV medication. HIV was by some described as being hidden in the body, even though it was not detectable. The participants described that it was vital for them to take responsibility for their HIV medication, as it kept the virus levels undetectable.

I do not actually have HIV anymore as long as I take the pills. I’m almost down to zero (viral load). (20)

Generally, ageing was associated with the need for medication, making the need for HIV medication non-deviant in old age. However, reflecting on how HIV medications affect the body, especially for those who had been taking medication for a long time, could foster anxiety. They expressed concerns about the potential inability to manage their HIV medication if they felt weak or disoriented, thereby having to depend on others for care.

#### Socio-emotional impact on everyday life

This theme describes older adults’ experiences of the socio-emotional impact of HIV on their everyday lives and interactions with others. HIV was described as “special” (19) compared to other diseases, mainly because of how other people think of and perceive HIV.

I have no problem with my HIV, but my problem lies with preconceived notions, either from individuals or from others attitude toward HIV—that is my problem. (14)

Older adults described both fear and experiences of prejudice from others, such as family, friends, or the public. They expressed encountering attitudes related to a lack of knowledge about HIV and how HIV is transmitted. Thoughts were represented in the absence of information on HIV, causing preconceived notions.

If you go out on the street and ask someone if they know what HIV is, I do not know if they know what it is. These days, you never hear about it (HIV) in newspapers or the media or anything; it is so hidden in a way//It is as if it (HIV) does not exist in society. (1)

Participants described their expectations of being judged by others and feelings of shame were experienced.

It (HIV) is still so shameful that no one wants to mention it. (9)

Being met with stereotypical perceptions about HIV associated with substance abuse, sexual orientation, or sexual intercourse were commonly described. Experiences of such attitudes seemed more pronounced for people living in smaller places or the countryside of Sweden or abroad. The fear of being judged by others influenced the decision to disclose their HIV status. There were different approaches to how open one should be about HIV, which was not always an easy decision. Some stated that they had chosen to be completely open to others about their HIV status, such as society and family. However, this level of openness seemed to be an exception. Some felt forced to disclose their HIV status even though they did not need it. This created contradictory feelings about whether to tell. Living with undetectable HIV was experienced as liberating. It was considered crucial to have autonomy in deciding when and to whom to disclose their HIV status, even though rules of conduct or other individuals also could influence their decisions.

I cannot go out and be open about my HIV status because it would hurt them (family) terribly. (22)

It was perceived as easier to talk about diseases other than HIV. For some HIV was being kept as a secret, and some only disclosed HIV to the closest ones.

No one knows, only my partner and myself. Nonetheless, of course, I have a doctor and nurse. Well, otherwise, it (HIV) is not discussed. It is ‘hush-hush. (18)

Participants described having a sense that others would pity them or that they would burden others with their HIV. Some indicated that there were no reasons to tell others about living with HIV as it would not lead to anything positive.

What should they know then? I have not told them’; there is no need to say to them. Individuals with different illnesses cannot tell everyone … (7)

Telling others about HIV was related to fear of rejection when disclosing HIV, often grounded in negative experiences. The fear of rejection was a possible barrier in meeting a new partner. For some, it was assumed that it was easier to find a partner living with HIV. Therefore, some chose to live alone, others voluntarily, and others did so involuntarily.

Many felt supported when they had experiences of disclosing their HIV status to family or friends and being met without prejudice. Those with the experience of being able to openly discuss HIV without fear of being judged found it reliable and essential. While some experienced support from HIV organizations, others felt that HIV organizations were not suitable for everyone or that there was no need to meet others living with HIV.

Even if there was no risk of HIV transmission, some participants described that they still were not able to eliminate the fear of being a potential transmitter. This could manifest as fear limiting social relations in everyday life and result in extra care to avoid the risk of transmitting HIV, even when having undetectable virus levels.

I have been given assurances, and I have asked that if I hold a grandchild in my arms, it (HIV transmission) cannot happen and that there would be any situation where I would transmit it to the child. (3)

This fear was experienced as a limitation to starting new intimate relationships. Others no longer considered it problematic as it was presumed to be more restrictive for them when they were young. The risk of potential HIV transmission did not only limit intimate relationships but could also limit relationships with friends, colleagues, or family members. From their past experiences, they learned that others sometimes saw a person living with HIV as a risk of transmitting HIV.

## Discussion

The findings revealed the multifaceted nature of ageing when living with HIV and underscored the importance of a nuanced understanding of the intertwining dynamics between HIV, ageing, and health. The older adults in this study described good health and well-being, although living with HIV was sometimes challenging. This aligns with previous research indicating that individuals living with HIV in Sweden report a relatively high quality of life. However, certain factors can negatively impact their lives (Zeluf-Andersson et al., 2018). The current study showed that some of these challenges were age-related. A scoping review on ageing with chronic diseases other than HIV showed that limitations associated with old age and psychological problems are common, together with difficulties in mobility, poor cognitive function, falls and incidents, wounds and injuries, undernutrition, and communication problems (Maresova et al., 2019). This scoping review concluded that preventing the negative consequences of chronic diseases and other age-related limitations requires multicomponent interventions. The findings of this study underscore the importance of acknowledging and strengthening patients´ resources to enhance well-being. There is a necessity to adopt a holistic approach to health, rather than solely focusing on the medical aspects of a disease. This study provides essential insights that can inform policy and practice, emphasizing the need to develop comprehensive support systems for older adults living with HIV. By integrating these findings, policymakers and practitioners can create more effective interventions that address both the physical and psychosocial aspects of ageing with HIV, ultimately leading to improved health outcomes and quality of life for this population.

Participants in the present study expressed the importance of filling everyday life with meaning, engaging in various activities, and maintaining social relationships with family and friends. Their well-being experiences were similar to those described by Dahlberg et al. (2009), who pointed out that well-being encompasses a sense of vitality to seize life opportunities. Their experiences of having a meaningful life was by the participants possible by being on ART. In Sweden almost all people living with HIV in receive ART, with the majority with well-adjusted HIV treatment (Gisslén et al., 2017). A well-adjusted HIV treatment has emerged as essential, decreasing physical restraints and thus influencing the ability to manage everyday activities. Even though the interviews in this study did not include questions regarding medical treatment and viral levels, participants stressed the significance of knowing that HIV was no longer transmittable. Receiving accurate information regarding infectiousness and rules of conduct has been stressed as significant, for instance, by women living with HIV in Sweden (Carlsson-Lalloo et al., 2021) and confirmed by men who have sex with men in Sweden (Herder & Agardh, 2019). These men also perceived inconsistencies in communication and information regarding infectiousness and rules of conduct and guidelines, and there seemed to be an absence of routines for communicating information (Herder & Agardh, 2019). Thus, providing people living with HIV access to information continuously, also when living with HIV for many years, is crucial for fostering a sense of security regarding treatment and non-transmittable HIV.

One challenge experienced by the participants was HIV-related stigma. HIV is associated with negative attitudes and stigmatization reflected in stereotypes, discrimination, and prejudices (Earnshaw & Chaudoir, 2009; Florom-Smith & De Santis, 2012; Reinius et al., 2021). Additionally, people living with HIV who are born abroad, inject drugs, have trans experience, are older, or belong to a sexual minority can be at risk of being exposed to multiple forms of stigmatization (Hsieh et al., 2022). This study illustrates how daily life encompasses various forms of stigmatization related to and beyond HIV, contributing to the experiences of multiple stigmatizations. One factor that can increase stigmatization is the negative and stereotypical perception of ageing, known as ageism. Ageism in healthcare is related to discrimination and age stereotypes that restrict access to health services (Araújo et al., 2023). Ageism can negatively impact health, life expectancy, and well-being and has far-reaching economic consequences for individuals and society (World Health Organization, 2021). Older adults living with HIV are at risk of being exposed to multiple stigmatizations, such as ageism and HIV-related stigma, leading to discrimination (Emlet, 2006). Therefore, it is crucial to be aware of and identify the risk of multiple stigmatizations in older adults living with HIV. The revised global targets set by UNAIDS aim to ensure that 95% of those living with HIV have a good health-related quality of life and zero discrimination (UNAIDS, 2020). Interventions are required to achieve this goal. More knowledge and an increase in awareness of HIV can enable proper treatment and quality care and prevent stigmatization and negative attitudes towards older adults living with HIV.

Additionally, the participants reported that HIV had a socio-emotional impact on their everyday lives. Thus, HIV has psychosocial consequences, particularly affecting social relationships with others. HIV being a burden with implications for relationships has previously been described by women living with HIV, showing that HIV can be a physical and mental barrier in various relationships (Carlsson-Lalloo et al., 2018). Living with HIV is linked to the risk of loneliness, which increases with age, as found in the current study. People living with HIV who report feelings of loneliness are more likely to report depression, alcohol, and tobacco use, and have fewer relationships (Greene et al., 2018). Hence, addressing social relationships as support for better health and well-being is essential.

### Methodological considerations—strengths and limitations

The rigour of thematic analysis can be discussed regarding reflexivity, credibility, and transferability (Sundler et al., 2019). Reflectivity involves having a reflective and open stance, being curious about participants’ experiences, and questioning one’s own preconceptions during interviews and analyses. Credibility relates to both the methodology and presentation of findings. After the first two interviews the research team transcribed the interviews immediately and assessed the quality and the interview questions to ensure that the interviewers captured the explored phenomenon. The analysis was conducted inductively and processed and discussed among all authors to ensure its trustworthiness, focusing on the experiences expressed by the interviewees. The analysis is presented thoroughly to make the methodology transparent to the reader. Quotes were carefully selected to illustrate the meanings in the results without allowing the identification of individual participants. Transferability involves relevance and usefulness, whether the findings are understandable and relevant to other contexts. A relatively large number of participants, with different characteristics and varied living conditions and life experiences were interviewed. The credibility of this study is grounded both in the variety of participants and the richness of their descriptions of lived experiences presented in the interviews. We found the qualitative approach that was used valuable to get in-depth insights on lived experiences of the phenomena in study.

However, a limitation of this study was the absence of individuals with experience in municipal care and support. The older adults recruited were considered relatively healthy, as for example all participants could with different aids, such as hearing aid, manage to be interviewed. Overall, older adults are underrepresented in research. This may be due to the challenges of including older individuals, especially those with disabilities or other vulnerabilities (Zeluf-Andersson et al., 2018). Dementia and mental health issues may be reasons for the underrepresentation of older adults in surveys and research (Sundler et al., 2019). The selection of interviewees may be biased, as those reporting well-being and good health may not represent individuals with more significant healthcare needs. To address this limitation, this study calls for further research to describe the needs of those with more substantial healthcare requirements.

Another limitation is that most of the participants in this study were native Swedish older adults. Despite the high prevalence of foreign-born people living with HIV in Sweden, 64%, in 2022 (Carlander & Mattsson, 2023), there is a lack of official sociodemographic data on country of birth for older adults living people with HIV in Sweden. As people living with HIV may experience multiple stigmatizations, this highlightes the need for further research in this area.

## Conclusions

The conditions of older adults living with HIV have changed. Although many older adults living with HIV experience positive health and well-being, various historically grounded challenges related to ageing and living with HIV persist. These findings advocate a holistic approach to health and well-being that goes beyond medical aspects and emphasizes the importance of a meaningful life with social relationships. HIV-related stigma can be a challenge encountered both in the public and healthcare as a consequence of insufficient knowledge about HIV and HIV transmission. Increased understanding of HIV and awareness of attitudes can prevent stigmatization and improve the quality of life of people living with HIV. These findings underscore the significance of individual resources and positive attitudes contributing to well-being, insights that can be used to inform policy and practices. Therefore, it is crucial to recognize and strengthen the individual resources and capacities of older adults living with HIV.
